# Magnetic Resonance Spectroscopy for Evaluating Portal-Systemic Encephalopathy in Patients with Chronic Hepatic Schistosomiasis Japonicum

**DOI:** 10.1371/journal.pntd.0005232

**Published:** 2016-12-15

**Authors:** Ying Li, Lihong Mei, Jinwei Qiang, Shuai Ju, Shuhui Zhao

**Affiliations:** 1 Department of Radiology, Jinshan Hospital & Shanghai Medical College, Fudan University, Shanghai, China; 2 Department of Dermatology, Jinshan Hospital & Shanghai Medical College, Fudan University, Shanghai, China; George Washington University, UNITED STATES

## Abstract

Portal-systemic encephalopathy (PSE) is classified as type B hepatic encephalopathy. Portal-systemic shunting rather than liver dysfunction is the main cause of PSE in chronic hepatic schistosomiasis japonicum (HSJ) patients. Owing to lack of detectable evidence of intrinsic liver disease, chronic HSJ patients with PSE are frequently clinically undetected or misdiagnosed, especially chronic HSJ patients with covert PSE (subclinical encephalopathy). In this study, we investigated whether magnetic resonance spectroscopy (MRS) could be a useful tool for diagnosing PSE in chronic HSJ patients. Magnetic resonance (MR) T1-weighted imaging, diffusion-weighted imaging, and MRS were performed in 41 chronic HSJ patients with suspected PSE and in 21 age-matched controls. The T1 signal intensity index (T1SI) and apparent diffusion coefficient (ADC) value were obtained in the Globus pallidus. Liver function was also investigated via serum ammonia and liver function tests. Higher T1SI and ADC values, increased lactate and glutamine levels, and decreased myo-inositol were found in the bilateral Globus pallidus in chronic HSJ patients with PSE. No significantly abnormal serum ammonia or liver function tests were observed in chronic HSJ patients with PSE. On the basis of these findings, we propose a diagnostic procedure for PSE in chronic HSJ patients. This study reveals that MRS can be useful for diagnosing PSE in chronic HSJ patients.

## Author Summary

Portal-systemic encephalopathy (PSE) is classified as type B hepatic encephalopathy. Portal-systemic shunting rather than liver dysfunction is the main cause of PSE in chronic hepatic schistosomiasis japonicum (HSJ) patients. Chronic HSJ patients with PSE are frequently misdiagnosed as suffering from dementia, depression, and other psychiatric disorders. However, there are few studies of this type of encephalopathy. In this study, magnetic resonance spectroscopy (MRS) was used as a noninvasive tool to detect abnormalities in brain metabolism in chronic HSJ patient with PSE. The results demonstrate that MRS would be useful for the diagnosis of PSE in chronic HSJ patients. Based on the results, a diagnostic procedure for PSE has also been proposed.

## Introduction

Portal-systemic shunting is formed subsequent to portal hypertension, which is a common occurrence in patients with liver cirrhosis and can lead to neuropsychiatric abnormalities [1, 2]. Portal-systemic encephalopathy (PSE) is classified as type B hepatic encephalopathy, which is an important complication of chronic liver cirrhosis and/or portal-systemic shunting [2, 3].

Portal-systemic shunting is the main cause of PSE in hepatic schistosomiasis japonicum (HSJ) patients [4]. These patients were infected with Schistosoma japonicum many years previously, resulting in hepatopathy of varying severity at that time. Although the active HSJ was completely cured, one of the sequelae, i.e., portal-systemic shunting persisted. These allegedly cured chronic HSJ patients lack biochemical evidence of intrinsic liver disease [5]; consequently, they do not receive suitable clinical management. Therefore, many patients develop PSE due to the portal-systemic shunting, which is frequently misdiagnosed as dementia, depression, or other psychiatric disorder [1]. Furthermore, chronic HSJ patients with covert PSE are usually clinically undetectable [1, 6].

The pathophysiology of PSE is not completely understood. It appears to be similar to hepatic encephalopathy, in which elevated ammonia and brain edema are widely recognized as the significant changes [7]. Magnetic resonance spectroscopy (MRS) has been used to detect abnormal metabolism in the brain. Previous studies showed increased glutamine levels in patients with hepatic encephalopathy [6, 8]. Recently, abnormal oxidation and carbohydrate fermentation were also found [9, 10]. However, to the best of our knowledge, no studies have investigated changes in brain metabolism in PSE. In this study, we used MRS as a noninvasive tool to detect metabolic abnormalities in the brains of chronic HSJ patients with PSE. Further, the goals of this study were to propose an appropriate diagnostic procedure for PSE in chronic HSJ patients.

## Methods

### Ethics statement

This study was approved by the Institutional Review Board of Jinshan Hospital, and written informed consent was obtained in all cases prior to magnetic resonance imaging (MRI) and laboratory tests.

### Patients

From August 2014 to April 2016, 151 consecutive chronic HSJ patients were recruited from the gastroenterology department of Jinshan Hospital. Chronic HSJ was diagnosed on the basis of a history of schistosomiasis and typical liver CT (linear hyper dense calcifications) or ultrasonic findings (linear strong echoes) [11]. The diagnosis of portal-systemic shunting was based on abdominal CT scanning for abnormal shunting vessels [12]. Thirteen shunting vessels were identified, namely, coronary, gastroepiploic, esophageal, para-esophageal, short gastric, peri splenic, splenorenal, gastrorenal, paraumbilical, abdominal wall vein, retroperitoneal-paravertebral, mesenteric, and omental vessels. Overt PSE was diagnosed on the basis of clinical symptoms and conventional neurological examinations. Covert PSE was diagnosed using the Digit Symbol Test (DST) and Number Connection Test A (NCT-A). Patients with test scores greater than two standard deviations were considered as covert PSE [13].

The exclusion criteria were as follows: liver cirrhotic patients (n = 73), history of alcoholism (n = 8), hepatocellular carcinoma (n = 11), patients using any drugs having liver (n = 2) or central nervous system toxicity (n = 3), and patients with total parenteral nutrition (n = 1). Patients unable to complete the MRI or MRS scanning (n = 5) and patients unable to complete the neuropsychological tests (n = 7) were also excluded. Finally, 41 patients were evaluated (25 males, 16 females; aged 49 to 87 years; mean, 67.2 ± 9.1 years) and divided into three groups according to the diagnostic criteria: non-PSE (n = 10), covert PSE (n = 12), and overt PSE (n = 19). Twenty-one age-matched healthy volunteers served as controls. These volunteers were recruited from the local population attending for routine physical examination (12 males, 9 females; aged 48 to 81 years; mean, 63.1 ± 9.9 years).

### Liver function tests

After 6 to 8 hours of fasting, blood samples were collected on the same day prior to MRI scanning. Serum alanine aminotransferase (ALT), aspartate aminotransferase (AST), total bilirubin (TB), direct bilirubin (DB), prothrombin time (PT), albumin, and plasma ammonia tests were performed in all patients to evaluate liver function. Whole blood manganese was also measured. All the liver function tests were also performed in the controls.

### MRI studies

MRI was performed on a 3.0-Tesla scanner (Verio, Siemens, Erlangen, Germany). First, an axial spin-echo T1-weighted imaging (T1WI), a diffusion-weighted imaging (DWI) with a simultaneously generated apparent diffusion coefficient (ADC) map were performed. Then, a multi-voxel MRS was performed using a point-resolved spectroscopy (PRESS) sequence with TR of 2000 ms, TE of 30 ms, and FOV of 10 cm × 10 cm. After automatic shimming, the spectral acquisition was performed with 256 averages.

### MRI data acquisition and processing

All the data were processed on a workstation using the MR manufacturer’s software. A 0.2 cm^2^-sized region of interest (ROI) was placed in the bilateral Globus pallidus on TIWI and ADC map. T1 signal intensity index (T1SI) and ADC value were measured three times and averaged. T1SI were calculated as follows: mean signal intensity divided by standard deviation (SD) of noise.

Spectral reconstruction was performed and spectral quality was assessed by measuring the full-width half maximum. The signal-to-noise ratio was considered acceptable when the amplitude of metabolite peaks was three times greater than that of the background noise. The resonance peak integrals of lactate (Lac, 1.33 ppm), N-acetyl aspartate (NAA, 2.01 ppm), glutamine (Glx, 2.15–2.45ppm), creatine (Cr, 3.02 ppm), choline (Cho, 3.22 ppm), and myo-inositol (mI, 3.55 ppm) were obtained. Cr peak was used as an internal reference, and the ratios of Lac/Cr, NAA/Cr, Glx/Cr, Cho/Cr, and mI/Cr were calculated.

### Statistical analysis

The sample size was estimated using a clinical research sample size calculator (CRESS V1.3). Statistical analyses were performed using SPSS 16.0 for Windows (SPSS Inc., Chicago, IL, USA). A nonparametric Kruskal-Wallis H-test was used to evaluate the differences in the ADC value and TISI among groups. One way ANOVA with a Bonferroni correction was used for liver function tests and metabolite ratios among groups. All values are represented as the mean ± standard deviation. A *p* value less than 0.05 was considered statistically significant.

## Results

### Clinical and laboratory findings

All patients had a history of HSJ more than 30 years ago. No active HSJ was found in any of these patients, nor was any upper gastrointestinal bleeding found within three months in any patients. Deficits in attention (n = 9, 21.9%), disturbance of sleep (n = 7, 17%), logopathy (n = 3, 7.3%), rigidity (n = 9, 21.9%), tremor (n = 6, 14.6%), and bradykinesia (n = 8, 19.5%) were observed in overt PSE. Liver function tests, magnetic resonance imaging, and spectroscopy findings in the different groups are listed in Table 1. No significant differences in any parameters were found between chronic HSJ patients without PSE and the controls. Increased DST (*p* < 0.05) and decreased NTC-A (*p* < 0.05) were observed in covert PSE group compared with the controls. No significant differences in ALT, AST, TB, DB, PT, and albumin or plasma ammonia levels were detected among the groups. Overt PSE and covert PSE patients showed higher blood manganese levels compared to non-PSE patients and controls (all *p*<0.001).

**Table 1:** Laboratory, magnetic resonance imaging, and spectroscopy findings in different groups

Parameter	Control (n = 21)	Non PSE (n = 10)	Covert PSE (n = 12)	Overt PSE (n = 19)	*P*
Age (y)	64.9±11.1	66.7±8.7	65.5±8.8	68.6±9.8	0.35
DST	49.49±8.34	47.35±7.61	28.59±5.8*	-	<0.001
NTC-A (s)	29.66±4.45	30.92±6.67	53.85±6.99**	-	<0.001
ALT (IU/L)	28.7±13.8	31.4±16.1	27.9±16.5	32.4±15	0.82
AST (IU/L)	44.3±8.9	35.5±10.7	39.3±19	48.7±17.6	0.1
TB (μmol/L)	23.8±12.8	22.6±7.3	21.8±9.4	22.8±12	0.97
UB (μmol/L)	10.7±5.2	8.8±3.1	9.5±3.5	9.6±4.1	0.69
PT (s)	11.6±1.3	11.8±1.1	11.8±1.2	11.8±2	0.97
Alb (g/L)	62.4±11.6	59.4±9.6	61±6.2	61.1±9.6	0.88
Am (μmol/L)	30.2±17.3	28.3±18.6	30.3±14.9	34.2±17.1	0.8
Mn (μg/L)	18.6±5.19	19.65±4.1	63.58±19***	74.08±29.84***	<0.001
T1SI	29.33±7.78	31.36±9.46	42.59±8.24*	55.85±11.31**	<0.001
ADC (×10–5 mm^2^/s)	82.15±2.78	85.99±5.39	91.27±6.89**	95.83±9.3**	<0.001
Lac/Cr	0.2±0.12	0.18±0.08	0.38±0.1*	1.17±1.37**	<0.001
NAA/Cr	1.7±0.11	1.7±0.15	1.71±0.23	1.69±0.21	0.99
Glx/Cr	0.4±0.13	0.36±0.15	0.47±0.16*	0.85±0.63**	<0.001
Cho/Cr	1.03±0.11	1.04±0.15	0.97±0.06	1.02±0.1	0.34
mI/Cr	0.52±0.15	0.49±0.14	0.42±0.13*	0.27±0.26**	<0.001

*p < 0.05

**p < 0.01

***p < 0.001

compared with controls. PSE: portal-systemic encephalopathy; DST: Digit Symbol Test; NCT-A: Number Connection Test A; ALT: alanine aminotransferase; AST: aspartate aminotransferase; TB: total bilirubin; DB: direct bilirubin; PT: prothrombin time; Alb: albumin; Am: ammonia; Mn: manganese; T1SI: T1 signal index; ADC: apparent diffusion coefficient; Lac/Cr: lactate/creatine; NAA/Cr: N-acetyl aspartate/creatine; Glx/Cr: glutamine/creatine; Cho/Cr: choline/creatine; mI/Cr: myo-inositol/creatine; A relatively stable Cr peak was used as an internal reference

### Imaging findings

**Fig 1:**
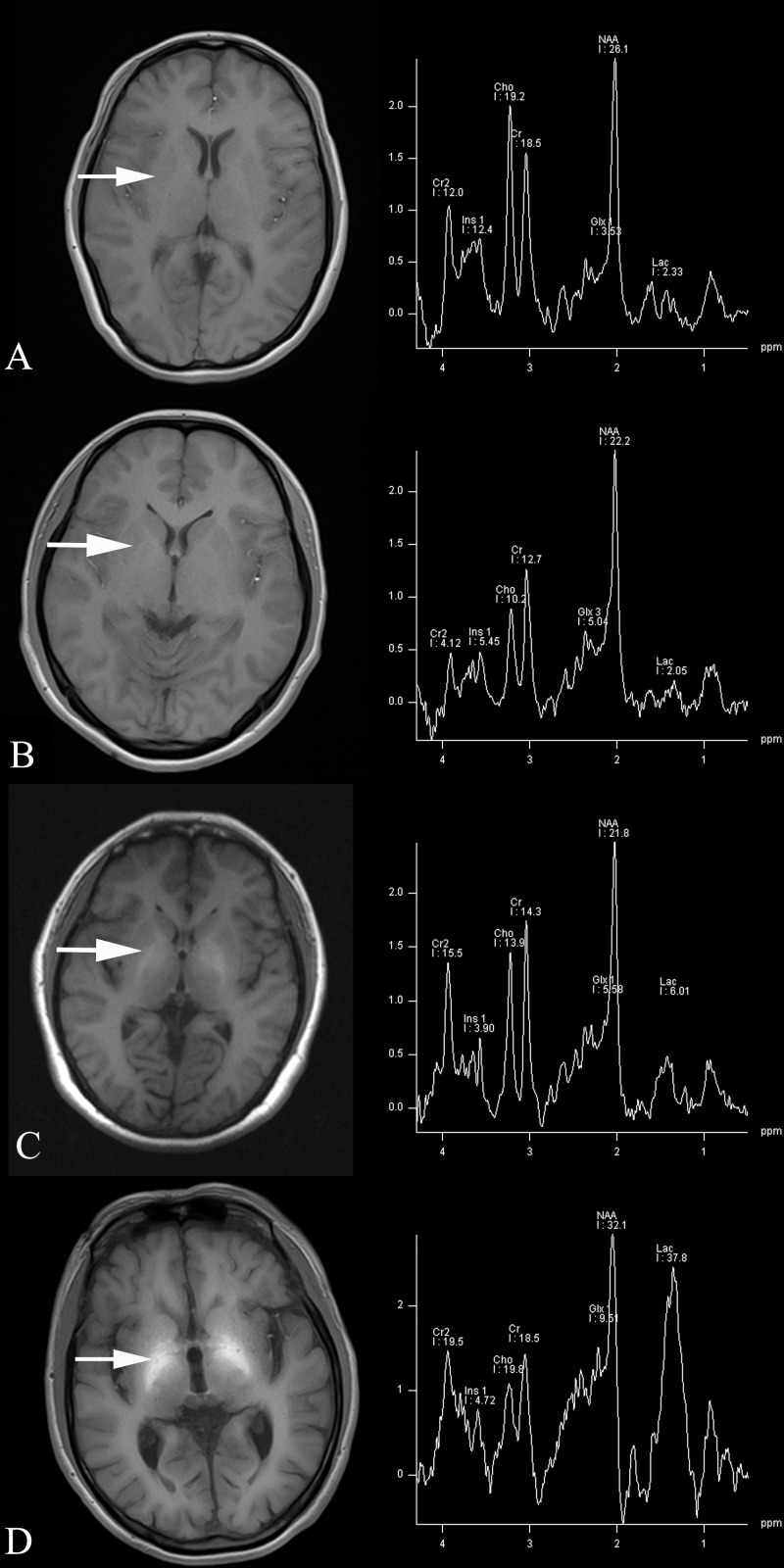
T1WI/MRS in the healthy volunteer and in chronic HSJ patients with and without PSE. No abnormal findings on T1WI and MRS were seen in a healthy volunteer (A) and a chronic hepatic schistosomiasis japonicum (HSJ) patient without portal-systemic encephalopathy (PSE) (B). Hyper intensity on T1WI, increased lactate (Lac/Cr) and glutamate/glutamine (Glx/Cr) levels, and decreased myo-inositol (mI/Cr) levels on MRS were found in the chronic HSJ patent with covert PSE (C). Obvious hyper intensity on T1WI, obviously increased lactate (Lac/Cr) and glutamate/glutamine (Glx/Cr) levels, and decreased myo-inositol (mI/Cr) levels on MRS were found in the chronic HSJ patient with overt PSE (D). The region of interest (ROI) was located in Globus pallidus on MRS.

**Fig 2:**
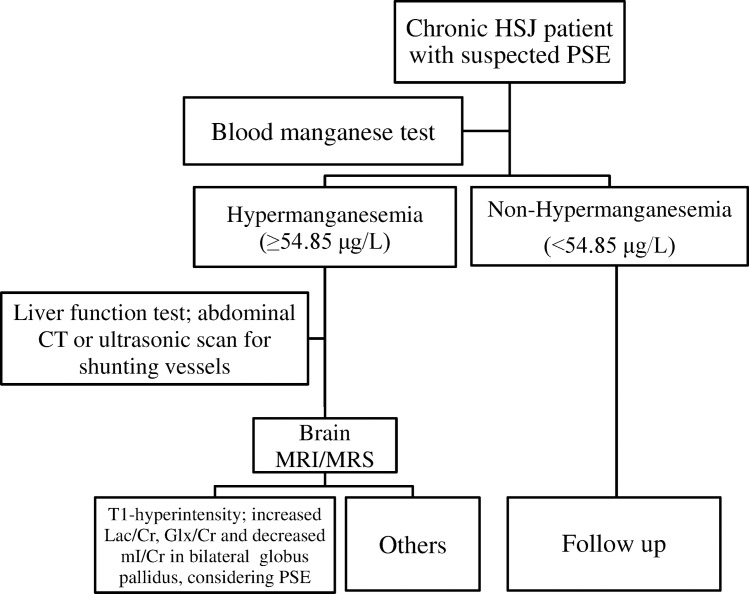
The diagnostic flow chart for chronic HSJ patient with suspected PSE. A fasted blood manganese should be tested in chronic HSJ patient with suspected PSE. If hypermanganesemia (blood manganese ≥ 54.85 μg/L) is found, liver function tests and abdominal CT or ultrasonic scan should be performed to establish whether liver dysfunction or portal-systemic shunting (or both) exist. Finally, a brain MRI and MRS can be performed to detect whether T1-hyperintensity, increased Lac, Glx, and decrease mI can be observed in bilateral Globus pallidus. Chronic HSJ patient with increased Lac and Glx but decreased mI should be considered as PSE. Chronic HSJ patients with abnormal neurological appearance should be considered as having overt PSE. Chronic HSJ patients with normal neurological parameters but abnormal neuropsychological performance should be considered as having covert PSE. Other patients should be followed up.

T1-hyperintensity in the bilateral Globus pallidus was observed in one patient without PSE (1/10, 10%), five patients with covert PSE (5/12, 41.6%), and 17 patients with overt PSE (17/19, 89.4%). T1SI and ADC value in the Globus pallidus progressively increased with the severity of PSE, being significantly higher in covert PSE and overt PSE patients than in non-PSE patients and controls (*p* < 0.05). Lac/Cr and Glx/Cr ratios progressively increased and mI/Cr ratio progressively decreased with the severity of PSE; a significant difference was observed in covert PSE (*p* < 0.05) and overt PSE patients (*p* < 0.001) as compared to controls (Fig 1). Furthermore, MRS findings (increased Lac, Glx, and decrease mI) was used as a gold standard. Using ROC analysis, a cut-off value of 54.85 μg/L for blood manganese was yielded a sensitivity and specificity of 94.7% and 88.4%, respectively for identify HSJ patients with PSE (S1 Fig). Based on these findings, we have proposed a diagnostic procedure for PSE in chronic HSJ patients (Fig 2).

## Discussion

In this study, hyper intensity on T1WI, increased ADC value, increased Lac (Lac/Cr), increased Glx (Glx/Cr), and decreased mI (mI/Cr) were found in the bilateral Globus pallidus in HSJ patients with PSE.

Schistosomiasis remains a major public health problem in many developing countries. According to a previous report, schistosomiasis affects more than 230 million people worldwide; an estimated 0.8 million people are infected with Schistosoma japonicum in China [14]. Usually, chronic HSJ patients lack objective physical findings such as gynecomastia and vascular spider, and abnormal liver function tests, which are frequently found in cirrhotic patients [1, 2]. However, hyper intensity in bilateral Globus pallidus on T1WI and portal-systemic shunting vessels could be detected by brain MRI and abdominal CT, respectively [5, 12]. Most of the patients develop neurological abnormalities (overt PSE) in the later stage of chronic hepatic schistosomiasis. In the early stage, these abnormalities could only be detected by neuropsychological tests (covert PES). Consequently, the majority of chronic HSJ patients with PSE remain clinically undetected.

As a result of portal-systemic shunting, the liver fails to efficiently clear toxins from the gut. These toxins such as ammonia and manganese are consequently elevated in circulating blood [5, 15]. The hyperammonemia is considered as the most important pathogenic feature in type A and type C hepatic encephalopathy [2], whereas the hypermanganesemia rather than hyperammonemia was a major finding in chronic HSJ patients. Manganese can be transported across the blood-brain barrier and can accumulate in the brain. In this study, the patients had a history of HSJ for more than 30 years. Thus, they experienced long-term exposure to hypermanganesemia and a resulting excessive brain manganese deposition. These patients also showed elevated blood manganese levels and bilateral symmetric hyper intensity in the Globus pallidus on T1WI, which is the characteristic appearance in brain manganese deposition [16]. In addition, T1WI hyper intensity can be found in chronic HSJ patients both with and without overt symptoms, which is consistent with the observations in previous studies [5, 17].

Increased ADC values were found in bilateral Globus pallidus in chronic HSJ patients. Previous studies showed that increased ADC values were associated with chronic manganese toxicity in manganese-exposed welders; the associated brain manganese deposition was linked to cytotoxic edema [18]. In HSJ patients with PSE, the extrapyramidal symptoms and MRI findings were similar to those seen in welders [19]. Therefore, in this study, an elevation of ADC values in bilateral Globus pallidus was also considered to be a consequence of manganese deposition.

An MRS appearance with findings typical of brain nitrogen metabolic changes, i.e., increased Lac, increased Glx, and decreased mI was found in chronic HSJ patients with PSE. These changes reflect a complex metabolic mechanism that occurs as a consequence of elevated blood manganese and brain manganese deposition in chronic HSJ patients with PSE. Lac is the final product of glycolysis. A recent study demonstrated increased Lac plays a major role in brain edema in cirrhotic rats [20]. We found an increased level of Lac in both covert PSE and overt PSE patients. Glx is a product of ammonia metabolism; increase of Glx is a major cause of brain edema in hepatic encephalopathy [21]. Studies on manganese neurotoxicology demonstrated that manganese disrupts brain energy metabolism and leads to the elevation of Lac and Glx [22, 23]. The decrease of mI is thought to be an attempt to compensate for increased Glx levels [23]. A decrease of mI was found in welders exposed to manganese [19]. Similarly, a decrease of mI was also found in patients with PSE. All these abnormal metabolites contribute to the brain edema that presented as an elevated ADC value in Globus pallidus of PSE patients.

The liver function and serum ammonia is normal in all chronic HSJ patients. Our results suggest that manganese may play the main role in the pathology of PSE in chronic HSJ patients. Slightly increased Lac/Cr and Glx/Cr, and slightly decreased mI/Cr were found in covert PSE. Obviously increased Lac/Cr and Glx/Cr, and obviously decreased mI/Cr were found in covert PSE. The results suggested that MRS had a potential benefits for the earlier identification of these patients.

### Limitations

This study has some limitations. First, glutamate and glutamine could not be distinguished on 3.0T MRI. Thus, we used Glx to represent both glutamate and glutamine. According to previous studies, excessive brain glutamine was found in hepatic encephalopathy [6]. Second, we did not compare the differences in metabolites on MRS between chronic HSJ patients with PSE and patients with hepatic encephalopathy. Third, a larger sample size is needed to validate the cut-off values of metabolites between non-PSE and PSE and covert and overt PSE in chronic HSJ patients.

## Conclusions

The present study demonstrated elevated Lac and Glx and decreased mI in the bilateral Globus pallidus in chronic HSJ patients with PSE. Thus, MRS can be a useful noninvasive tool for the diagnosis of PSE in chronic HSJ patients.

Chemical Abstracts Service (CAS) Number

Ammonia: 7664-41-7
Choline: 123-41-1
Creatine: 57-00-1
Glutamate: 56-86-0
Glutamine: 56-85-9
Lactate: 50-21-5
Manganese: 7439-96-5
N-acetyl aspartate: 2545-40-6
Myo-inositol: 87-89-8

## Supporting Information

S1 Checklist Standards for Reporting Diagnostic accuracy studies. (PDF)

S1 Table The levels of liver function, blood manganese, TISI, ADC, MRS and neurological tests. alanine aminotransferase (ALT), aspartate aminotransferase (AST), total bilirubin (TB), direct bilirubin (DB), prothrombin time (PT), albumin (Alb), ammonia (Am), manganese (Mn), lactate (Lac), N-acetyl aspartate (NAA), glutamine (Glx), creatine (Cr), choline (Cho), myo-inositol (mI), T1 signal intensity index (T1SI), apparent diffusion coefficient (ADC), Digit Symbol Test (DST), Number Connection Test A (NCT-A).
(DOCX)

S1 Fig The ROC curve of the blood manganese for predicting PSE. Using ROC analysis, a cut-off value of 54.85 μg/L for blood manganese yielded a sensitivity and specificity of 94.7% and 88.4%, respectively for identify HSJ patients with PSE.
(TIF)

S1 Flow Diagram Prototypical STARD diagram to report flow of participants through the study (PDF)
