# Supplementary material for: Magnetic Resonance Spectroscopy for Evaluating Portal-Systemic Encephalopathy in Patients with Chronic Hepatic Schistosomiasis Japonicum
Source: PLoS Negl Trop Dis. 2016 Dec 15;10(12):e0005232. doi: 10.1371/journal.pntd.0005232 (PMC5199111; doi:10.1371/journal.pntd.0005232)
Supplement: S1 Table — alanine aminotransferase (ALT), aspartate aminotransferase (AST), total bilirubin (TB), direct bilirubin (DB), prothrombin time (PT), albumin (Alb), ammonia (Am), manganese (Mn), lactate (Lac), N-acetyl aspartate (NAA), glutamine (Glx), creatine (Cr), choline (Cho), myo-inositol (mI), T1 signal intensity index (T1SI), apparent diffusion coefficient (ADC), Digit Symbol Test (DST), Number Connection Test A (NCT-A). (DOCX) [file pntd.0005232.s002.docx]

Supplementary table 1 The levels of liver function, blood manganese, TISI, ADC, MRS and neurological tests.

| Num | Group | Sex | Age (y) | ALT (IU/L) | AST (IU/L) | TB (μmol/L) | UB (μmol/L) | PT (s) | Alb (g/L) | Am (μmol/L) | Mn (μg/L) | Lac/Cr | Glx/Cr | MI/Cr | NAACr | Cho/Cr | T1SI | ADC (×10-5 mm2/s) | DST | NTC-A (s) |
| --- | --- | --- | --- | --- | --- | --- | --- | --- | --- | --- | --- | --- | --- | --- | --- | --- | --- | --- | --- | --- |
| 1 | normal | M | 55.00 | 46.00 | 37.00 | 43.40 | 5.70 | 10.50 | 73.00 | 48.00 | 23.80 | 0.09 | 0.40 | 0.53 | 1.82 | 0.89 | 31.30 | 80.00 | 42.94 | 29.01 |
| 2 | normal | F | 67.00 | 45.00 | 37.00 | 3.50 | 0.30 | 10.40 | 64.00 | 15.00 | 18.40 | 0.26 | 0.52 | 0.33 | 1.80 | 1.11 | 36.58 | 78.50 | 54.30 | 30.52 |
| 3 | normal | M | 75.00 | 24.00 | 50.00 | 49.70 | 16.30 | 11.80 | 68.00 | 23.00 | 22.40 | 0.16 | 0.33 | 0.48 | 1.70 | 1.19 | 35.12 | 87.00 | 46.86 | 30.64 |
| 4 | normal | F | 50.00 | 29.00 | 48.00 | 10.70 | 15.10 | 12.30 | 51.00 | 20.00 | 15.10 | 0.13 | 0.40 | 0.58 | 1.80 | 0.92 | 35.93 | 83.80 | 39.22 | 29.42 |
| 5 | normal | M | 62.00 | 17.00 | 45.00 | 14.30 | 12.20 | 11.30 | 78.00 | 19.00 | 16.80 | 0.13 | 0.61 | 0.66 | 1.85 | 1.05 | 30.08 | 85.00 | 59.01 | 22.10 |
| 6 | normal | M | 58.00 | 41.00 | 34.00 | 45.20 | 18.60 | 13.80 | 44.00 | 21.00 | 22.70 | 0.01 | 0.18 | 0.33 | 1.77 | 1.09 | 38.00 | 81.00 | 51.64 | 22.86 |
| 7 | normal | F | 61.00 | 10.00 | 36.00 | 27.10 | 7.40 | 9.70 | 63.00 | 9.00 | 26.20 | 0.06 | 0.47 | 0.51 | 1.76 | 0.90 | 39.51 | 80.05 | 48.95 | 33.23 |
| 8 | normal | F | 81.00 | 11.00 | 58.00 | 21.60 | 9.10 | 11.70 | 75.00 | 5.00 | 7.50 | 0.34 | 0.28 | 0.52 | 1.60 | 0.94 | 23.65 | 79.25 | 57.28 | 33.40 |
| 9 | normal | M | 62.00 | 45.00 | 40.00 | 17.00 | 11.80 | 12.00 | 46.00 | 17.00 | 11.50 | 0.30 | 0.47 | 0.43 | 1.83 | 0.96 | 16.64 | 85.40 | 42.29 | 33.94 |
| 10 | normal | F | 71.00 | 19.00 | 58.00 | 22.80 | 14.70 | 13.20 | 73.00 | 39.00 | 19.50 | 0.37 | 0.53 | 0.66 | 1.72 | 1.19 | 21.58 | 83.00 | 62.56 | 36.68 |
| 11 | normal | M | 84.00 | 25.00 | 49.00 | 5.00 | 13.90 | 10.40 | 71.00 | 62.00 | 24.60 | 0.22 | 0.40 | 0.33 | 1.60 | 1.18 | 22.32 | 79.65 | 49.46 | 34.37 |
| 12 | normal | F | 52.00 | 8.00 | 35.00 | 22.30 | 7.30 | 12.90 | 44.00 | 21.00 | 16.20 | 0.16 | 0.56 | 0.71 | 1.60 | 0.90 | 21.58 | 84.20 | 43.12 | 25.41 |
| 13 | normal | M | 69.00 | 39.00 | 40.00 | 14.30 | 0.70 | 9.80 | 68.00 | 39.00 | 14.80 | 0.20 | 0.11 | 0.57 | 1.71 | 1.17 | 18.13 | 80.03 | 62.84 | 27.06 |
| 14 | normal | M | 62.00 | 21.00 | 52.00 | 25.90 | 18.00 | 13.20 | 69.00 | 52.00 | 25.90 | 0.47 | 0.48 | 0.33 | 1.69 | 1.07 | 23.55 | 78.00 | 42.00 | 27.37 |
| 15 | normal | F | 58.00 | 28.00 | 56.00 | 11.90 | 11.70 | 9.40 | 58.00 | 45.00 | 13.10 | 0.01 | 0.28 | 0.65 | 1.78 | 1.18 | 17.35 | 81.60 | 61.97 | 22.97 |
| 16 | normal | F | 48.00 | 19.00 | 36.00 | 32.00 | 8.70 | 11.20 | 82.00 | 50.00 | 23.20 | 0.37 | 0.54 | 0.63 | 1.61 | 0.98 | 23.94 | 81.75 | 39.18 | 34.96 |
| 17 | normal | M | 67.00 | 47.00 | 28.00 | 30.80 | 8.80 | 12.30 | 61.00 | 36.00 | 20.80 | 0.12 | 0.34 | 0.23 | 1.35 | 1.07 | 33.28 | 84.35 | 45.46 | 25.67 |
| 18 | normal | M | 85.00 | 8.00 | 53.00 | 31.70 | 6.20 | 11.70 | 48.00 | 17.00 | 22.50 | 0.23 | 0.38 | 0.60 | 1.71 | 0.99 | 39.16 | 87.80 | 59.53 | 35.31 |
| 19 | normal | F | 52.00 | 43.00 | 38.00 | 15.80 | 8.20 | 13.40 | 56.00 | 18.00 | 18.50 | 0.15 | 0.39 | 0.47 | 1.65 | 0.91 | 37.16 | 81.55 | 40.16 | 25.94 |
| 20 | normal | M | 64.00 | 44.00 | 51.00 | 36.70 | 17.50 | 11.30 | 67.00 | 61.00 | 13.20 | 0.20 | 0.30 | 0.86 | 1.73 | 0.91 | 38.06 | 83.45 | 50.51 | 29.26 |
| 21 | normal | M | 79.00 | 33.00 | 50.00 | 17.50 | 11.80 | 11.70 | 52.00 | 17.00 | 13.80 | 0.19 | 0.37 | 0.57 | 1.63 | 1.09 | 33.05 | 79.80 | 39.91 | 32.83 |
| 22 | non PSE | M | 62.00 | 12.00 | 18.00 | 21.00 | 9.90 | 13.00 | 54.00 | 52.00 | 18.50 | 0.20 | 0.36 | 0.48 | 1.80 | 1.13 | 36.37 | 83.05 | 39.03 | 35.78 |
| 23 | non PSE | F | 82.00 | 16.00 | 31.00 | 15.70 | 7.90 | 12.00 | 52.00 | 25.00 | 24.00 | 0.15 | 0.26 | 0.47 | 1.69 | 1.00 | 44.34 | 84.68 | 44.66 | 35.48 |
| 24 | non PSE | M | 50.00 | 16.00 | 35.00 | 20.50 | 9.00 | 10.20 | 53.00 | 16.00 | 20.90 | 0.14 | 0.36 | 0.45 | 1.72 | 1.11 | 22.80 | 81.63 | 54.30 | 21.36 |
| 25 | non PSE | F | 61.00 | 58.00 | 36.00 | 20.00 | 6.30 | 13.00 | 63.00 | 18.00 | 24.20 | 0.05 | 0.32 | 0.39 | 1.63 | 0.80 | 32.46 | 83.86 | 58.34 | 20.71 |
| 26 | non PSE | M | 70.00 | 41.00 | 29.00 | 13.80 | 6.60 | 11.60 | 67.00 | 51.00 | 20.70 | 0.18 | 0.21 | 0.60 | 1.40 | 1.09 | 40.41 | 80.53 | 56.05 | 37.18 |
| 27 | non PSE | F | 70.00 | 17.00 | 31.00 | 25.80 | 14.20 | 11.30 | 57.00 | 12.00 | 18.70 | 0.32 | 0.38 | 0.64 | 1.81 | 0.95 | 33.47 | 95.15 | 40.37 | 36.16 |
| 28 | non PSE | M | 75.00 | 26.00 | 38.00 | 15.30 | 7.00 | 11.30 | 69.00 | 45.00 | 10.60 | 0.18 | 0.34 | 0.20 | 1.71 | 1.10 | 37.34 | 81.63 | 39.98 | 34.84 |
| 29 | non PSE | M | 66.00 | 36.00 | 48.00 | 26.70 | 4.10 | 10.30 | 54.00 | 48.00 | 17.30 | 0.22 | 0.37 | 0.60 | 1.61 | 0.84 | 43.77 | 95.41 | 52.73 | 23.85 |
| 30 | non PSE | F | 63.00 | 47.00 | 57.00 | 36.50 | 12.50 | 12.40 | 78.00 | 11.00 | 17.90 | 0.29 | 0.25 | 0.40 | 1.96 | 1.28 | 15.50 | 88.45 | 39.23 | 28.29 |
| 31 | non PSE | M | 68.00 | 45.00 | 32.00 | 30.50 | 10.90 | 13.30 | 47.00 | 5.00 | 23.70 | 0.08 | 0.74 | 0.65 | 1.65 | 1.12 | 45.16 | 85.50 | 48.82 | 35.51 |
| 32 | covert PSE | F | 61.00 | 58.00 | 36.00 | 22.56 | 7.76 | 13.00 | 63.00 | 18.00 | 78.40 | 0.24 | 0.46 | 0.29 | 1.70 | 0.95 | 40.09 | 89.50 | 33.96 | 60.00 |
| 33 | covert PSE | M | 62.00 | 12.00 | 18.00 | 23.59 | 11.93 | 13.00 | 54.00 | 52.00 | 71.20 | 0.46 | 0.47 | 0.32 | 1.47 | 0.88 | 57.71 | 97.75 | 25.76 | 62.00 |
| 34 | covert PSE | F | 66.00 | 16.00 | 31.00 | 18.06 | 9.68 | 12.10 | 52.00 | 25.00 | 83.30 | 0.21 | 0.28 | 0.39 | 1.95 | 1.02 | 49.17 | 88.85 | 34.48 | 55.58 |
| 35 | covert PSE | M | 49.00 | 16.00 | 35.00 | 22.56 | 10.50 | 10.20 | 53.00 | 16.00 | 61.30 | 0.42 | 0.52 | 0.40 | 1.50 | 0.94 | 43.74 | 80.00 | 20.52 | 41.19 |
| 36 | covert PSE | M | 60.00 | 41.00 | 29.00 | 17.29 | 7.95 | 11.60 | 67.00 | 51.00 | 80.10 | 0.35 | 0.46 | 0.31 | 2.15 | 1.04 | 50.44 | 96.30 | 32.85 | 54.63 |
| 37 | covert PSE | M | 70.00 | 17.00 | 31.00 | 29.26 | 15.84 | 11.30 | 57.00 | 12.00 | 82.90 | 0.43 | 0.45 | 0.40 | 1.81 | 0.90 | 41.36 | 99.15 | 34.81 | 59.34 |
| 38 | covert PSE | F | 67.00 | 31.00 | 67.00 | 8.62 | 5.01 | 11.20 | 57.00 | 37.00 | 69.10 | 0.28 | 0.45 | 0.43 | 1.64 | 1.00 | 43.38 | 102.70 | 24.77 | 57.56 |
| 39 | covert PSE | M | 70.00 | 12.00 | 16.00 | 10.90 | 5.20 | 11.50 | 62.00 | 41.00 | 31.30 | 0.47 | 0.38 | 0.40 | 1.65 | 0.90 | 46.67 | 84.50 | 34.74 | 48.50 |
| 40 | covert PSE | F | 67.00 | 34.00 | 55.00 | 16.44 | 8.02 | 10.10 | 70.00 | 17.00 | 73.80 | 0.31 | 0.46 | 0.38 | 1.39 | 1.03 | 42.35 | 84.50 | 23.06 | 40.93 |
| 41 | covert PSE | M | 85.00 | 17.00 | 30.00 | 43.31 | 13.03 | 14.60 | 65.00 | 43.00 | 47.00 | 0.38 | 0.31 | 0.39 | 1.74 | 1.05 | 62.66 | 90.50 | 32.41 | 59.13 |
| 42 | covert PSE | M | 58.00 | 57.00 | 79.00 | 18.45 | 5.50 | 11.70 | 64.00 | 14.00 | 54.50 | 0.51 | 0.53 | 0.55 | 1.96 | 1.00 | 41.78 | 86.60 | 20.28 | 54.74 |
| 43 | covert PSE | M | 71.00 | 24.00 | 44.00 | 30.45 | 13.23 | 11.60 | 68.00 | 38.00 | 30.10 | 0.51 | 0.91 | 0.78 | 1.54 | 0.93 | 31.76 | 94.85 | 25.40 | 52.60 |
| 44 | overt PSE | M | 66.00 | 26.00 | 46.00 | 13.71 | 12.04 | 9.50 | 69.00 | 16.00 | 55.20 | 0.36 | 0.81 | 0.18 | 1.56 | 1.19 | 47.22 | 82.85 |  |  |
| 45 | overt PSE | F | 57.00 | 26.00 | 46.00 | 23.86 | 9.14 | 13.50 | 59.00 | 33.00 | 34.80 | 0.21 | 0.84 | 0.19 | 1.94 | 1.02 | 40.09 | 86.75 |  |  |
| 46 | overt PSE | F | 83.00 | 26.00 | 31.00 | 9.16 | 5.71 | 12.30 | 66.00 | 39.00 | 56.70 | 0.47 | 0.96 | 0.23 | 1.70 | 0.90 | 68.41 | 96.45 |  |  |
| 47 | overt PSE | M | 55.00 | 17.00 | 30.00 | 44.45 | 13.49 | 14.60 | 65.00 | 43.00 | 95.50 | 1.21 | 0.56 | 0.02 | 1.45 | 1.00 | 54.95 | 94.00 |  |  |
| 48 | overt PSE | M | 66.00 | 26.00 | 46.00 | 34.42 | 9.48 | 9.50 | 56.00 | 17.00 | 92.00 | 0.89 | 1.06 | 0.26 | 1.56 | 0.97 | 67.02 | 84.65 |  |  |
| 49 | overt PSE | F | 62.00 | 26.00 | 46.00 | 35.72 | 8.04 | 13.50 | 69.00 | 33.00 | 72.90 | 2.22 | 1.15 | 0.29 | 1.54 | 1.08 | 51.92 | 87.65 |  |  |
| 50 | overt PSE | M | 70.00 | 62.00 | 66.00 | 11.54 | 5.14 | 11.50 | 63.00 | 41.00 | 55.70 | 1.23 | 0.46 | 0.00 | 1.41 | 1.07 | 50.83 | 96.75 |  |  |
| 51 | overt PSE | M | 71.00 | 24.00 | 44.00 | 30.23 | 13.69 | 11.60 | 68.00 | 38.00 | 84.10 | 0.92 | 0.24 | 0.00 | 1.58 | 1.13 | 50.52 | 96.50 |  |  |
| 52 | overt PSE | F | 67.00 | 34.00 | 55.00 | 16.64 | 8.88 | 10.10 | 70.00 | 26.00 | 61.60 | 1.29 | 0.76 | 0.23 | 1.52 | 1.07 | 55.04 | 105.30 |  |  |
| 53 | overt PSE | M | 87.00 | 31.00 | 67.00 | 8.20 | 5.72 | 11.20 | 57.00 | 37.00 | 62.30 | 1.19 | 0.55 | 0.02 | 1.98 | 0.90 | 61.44 | 100.15 |  |  |
| 54 | overt PSE | M | 58.00 | 57.00 | 79.00 | 19.30 | 4.21 | 11.70 | 64.00 | 14.00 | 50.30 | 0.42 | 0.15 | 0.87 | 2.04 | 0.92 | 59.18 | 110.90 |  |  |
| 55 | overt PSE | M | 69.00 | 24.00 | 20.00 | 14.89 | 5.89 | 11.90 | 71.00 | 48.00 | 92.90 | 1.04 | 1.70 | 0.13 | 1.50 | 1.06 | 62.64 | 88.25 |  |  |
| 56 | overt PSE | F | 53.00 | 66.00 | 51.00 | 8.81 | 5.85 | 12.30 | 66.00 | 11.00 | 57.50 | 0.27 | 0.61 | 0.58 | 1.99 | 0.92 | 38.00 | 105.50 |  |  |
| 57 | overt PSE | F | 65.00 | 16.00 | 35.00 | 23.66 | 11.27 | 11.10 | 53.00 | 16.00 | 43.80 | 0.43 | 0.37 | 0.62 | 1.41 | 1.18 | 44.88 | 91.90 |  |  |
| 58 | overt PSE | M | 82.00 | 38.00 | 69.00 | 41.27 | 17.66 | 10.90 | 43.00 | 67.00 | 43.20 | 0.27 | 0.35 | 0.28 | 1.74 | 1.04 | 50.57 | 84.50 |  |  |
| 59 | overt PSE | F | 75.00 | 16.00 | 35.00 | 22.73 | 11.92 | 11.10 | 35.00 | 56.00 | 97.90 | 5.22 | 2.91 | 0.03 | 1.73 | 0.96 | 52.02 | 96.75 |  |  |
| 60 | overt PSE | M | 65.00 | 20.00 | 21.00 | 10.06 | 5.29 | 10.10 | 67.00 | 67.00 | 143.50 | 0.13 | 0.48 | 0.47 | 1.88 | 1.20 | 53.71 | 91.60 |  |  |
| 61 | overt PSE | F | 70.00 | 42.00 | 70.00 | 22.73 | 11.02 | 10.10 | 66.00 | 31.00 | 71.50 | 0.31 | 0.90 | 0.70 | 1.88 | 0.92 | 66.22 | 105.40 |  |  |
| 62 | overt PSE | M | 82.00 | 38.00 | 69.00 | 41.04 | 17.83 | 17.90 | 53.00 | 17.00 | 136.20 | 4.21 | 1.24 | 0.06 | 1.75 | 0.94 | 86.58 | 115.00 |  |  |

alanine aminotransferase (ALT), aspartate aminotransferase (AST), total bilirubin (TB), direct bilirubin (DB), prothrombin time (PT), albumin (Alb), ammonia (Am), manganese (Mn) , lactate (Lac/Cr), N-acetyl aspartate (NAA/Cr), glutamine (Glx/Cr), creatine (Cr/Cr), choline (Cho/Cr), myo-inositol (mI/Cr), T1 signal intensity index (T1SI), apparent diffusion coefficient (ADC), Digit Symbol Test (DST), Number Connection Test A (NCT-A).
