# Supplementary figures and images for: Magnetic Resonance Spectroscopy for Evaluating Portal-Systemic Encephalopathy in Patients with Chronic Hepatic Schistosomiasis Japonicum
Source: PLoS Negl Trop Dis. 2016 Dec 15;10(12):e0005232. doi: 10.1371/journal.pntd.0005232 (PMC5199111; doi:10.1371/journal.pntd.0005232)

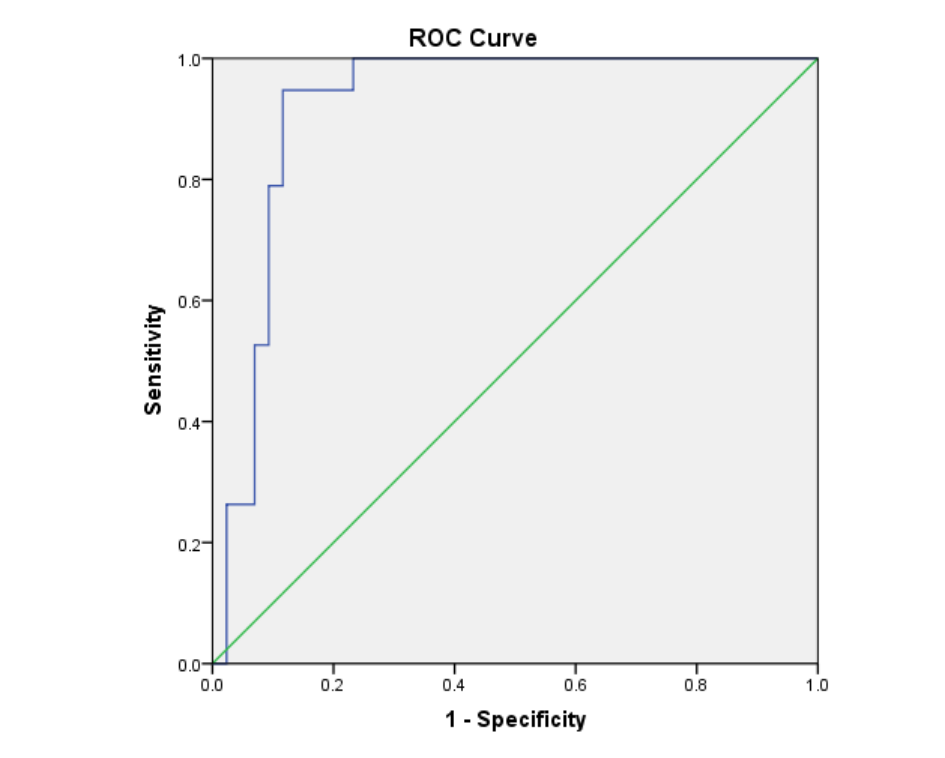

Supplement: S1 Fig — Using ROC analysis, a cut-off value of 54.85 μg/L for blood manganese yielded a sensitivity and specificity of 94.7% and 88.4%, respectively for identify HSJ patients with PSE. (TIF) [file pntd.0005232.s003.tif]

Prototypical STARD diagram to report flow of participants through the study

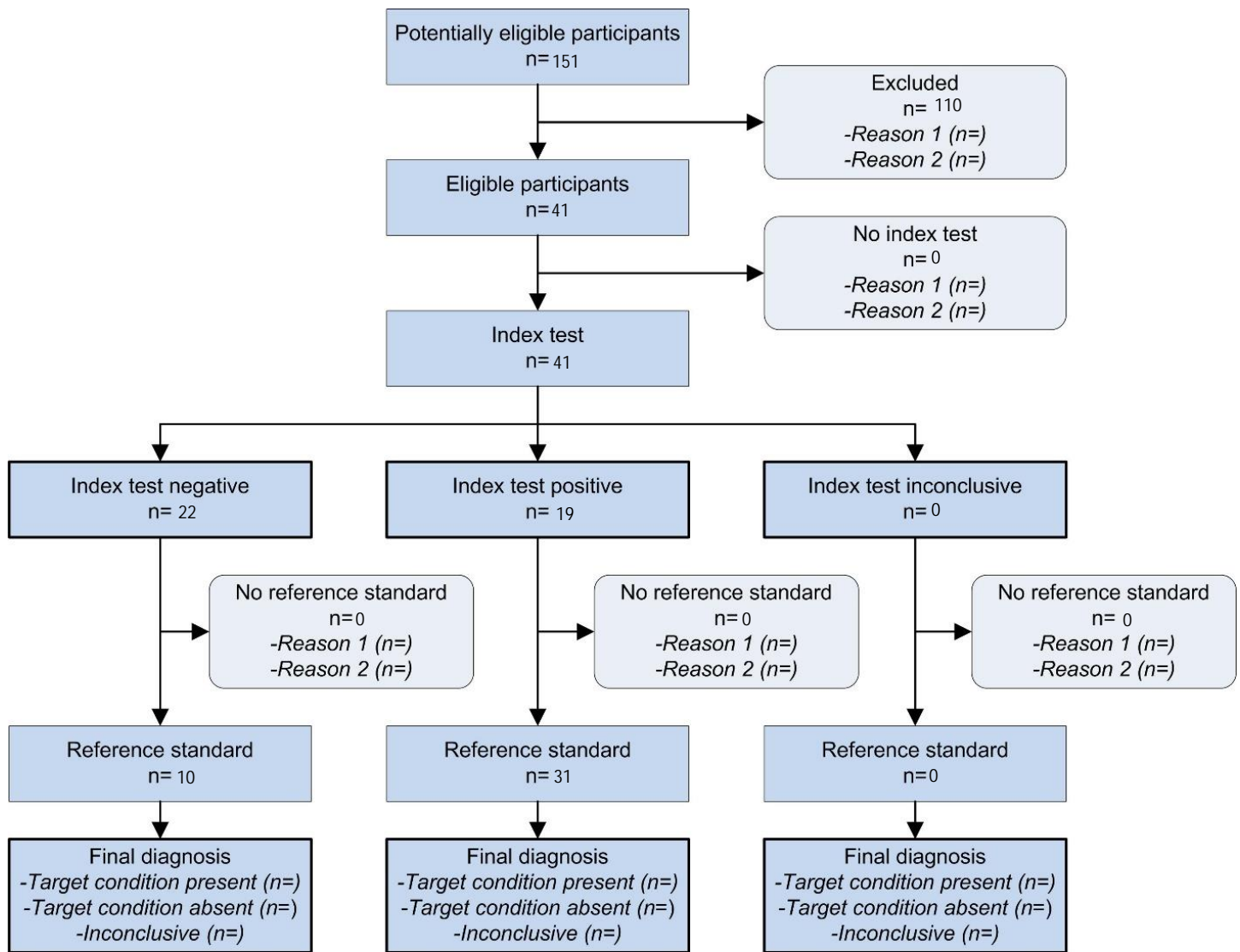

Supplement: S1 Flow Diagram — (PDF) [file pntd.0005232.s004.pdf]
